# Blood Biomarkers Predict the Cognitive Effects of Aripiprazole in Patients with Acute Schizophrenia

**DOI:** 10.3390/ijms18030568

**Published:** 2017-03-06

**Authors:** Hikaru Hori, Reiji Yoshimura, Asuka Katsuki, Kiyokazu Atake, Ryohei Igata, Yuki Konishi, Hiroki Beppu, Hirotaka Tominaga

**Affiliations:** Department of Psychiatry, University of Occupational and Environmental Health, Iseigaoka, Yahatanishi-ku, Kitakyushu, Fukuoka 8078555, Japan; yoshi621@med.uoeh-u.ac.jp (R.Y.); asuka-k@med.uoeh-u.ac.jp (A.K.); k-atake@med.uoeh-u.ac.jp (K.A.); igataryouhei@gmail.com (R.I.); konikoni0125@hotmail.co.jp (Y.K.); hirokibeppu0928@hotmail.co.jp (H.B.); inevitable_instinct77@yahoo.co.jp (H.T.)

**Keywords:** brain-derived neurotrophic factor, schizophrenia, aripiprazole, cognitive function

## Abstract

Aripiprazole has been reported to exert variable effects on cognitive function in patients with schizophrenia. Therefore, in the present study, we evaluated biological markers, clinical data, and psychiatric symptoms in order to identify factors that influence cognitive function in patients with schizophrenia undergoing aripiprazole treatment. We evaluated cognitive function in 51 patients with schizophrenia using Brief Assessment of Cognition in Schizophrenia (BACS), as well as background information, psychiatric symptoms, plasma catecholamine metabolites—homovanillic acid (HVA), 3-methoxy-4-hydroxyphenylglycol (MHPG)—, and serum brain-derived neurotrophic factor (BDNF). Multivariate analyses were performed in order to identify factors independently associated with cognitive function. Brain-derived neurotrophic factor levels, number of hospitalizations, and MHPG levels were associated with verbal memory and learning. Total hospitalization period and MHPG levels were associated with working memory. Age at first hospitalization and education were associated with motor speed. The number of hospital admissions, Positive and Negative Syndrome Scale negative subscale scores (PANSS-N), MHPG levels, BDNF levels, and Drug-Induced Extrapyramidal Symptoms Scale (DIEPSS) scores were associated with verbal fluency. Homovanillic acid and MHPG levels, duration of illness, and PANSS-N scores were associated with attention and processing speed. Brain-derived neurotrophic factor and MHPG levels were associated with executive function. These results suggest that treatment of psychiatric symptoms and cognitive dysfunction may be improved in patients treated with aripiprazole by controlling for these contributing factors.

## 1. Introduction

Aripiprazole has a unique pharmacology with partial agonist activity at dopamine D2/D3 receptors, associated with a low risk of hyperprolactinemia [1,2], and partial agonist activity at serotonin (5-HT) 5-HT1A receptors and antagonist activity at 5-HT2A receptors. It has a low risk of metabolic side effects, weight gain, increase in total cholesterol and blood pressure, hyperprolactinemia and sedation [3,4]. Therefore, it has widely been recommended as a first-line treatment for schizophrenia [5,6].

The actions of antipsychotic drugs on the catecholamine system, particularly the dopamine system, have led to many studies of catecholamine metabolites as possible markers for psychosis and the antipsychotic response. Plasma homovanillic acid (HVA) was shown to reflect central or brain dopamine activity based on studies in animals and humans [7,8,9]. Several studies of schizophrenia have noted that the behavioral response to antipsychotic drugs, such as a decrease in psychosis, parallels a decrease in plasma HVA levels in schizophrenic patients over time [10,11,12,13,14,15,16,17,18,19,20]. Atypical antipsychotics increase the plasma 3-methoxy-4-hydroxyphenylglycol (MHPG) levels and are associated with negative symptoms and cognitive functions [19,21,22]. Brain-derived neurotrophic factor (BDNF) is the most widely distributed neurotrophic factor in the brain, the levels of which have been associated with schizophrenia. The role of BDNF in cognition has also been shown for some animal and human schizophrenia [23,24,25,26].

Recent trends in the treatment of schizophrenia include the provision of recovery-oriented care, which highlights the need to improve cognitive and social functioning in order for each patient to achieve his or her treatment goals. Research has demonstrated that, unlike with other antipsychotics, the dose of aripiprazole has no significant effect on cognitive function or subjective well-being in patients with schizophrenia [27,28]. In addition, previous studies have reported that the dose of atypical antipsychotics such as risperidone and olanzapine is negatively correlated with cognitive function in patients with schizophrenia [27], and that dose reduction results in cognitive improvement in this patient population [29].

However, the effects of aripiprazole on cognitive function are reported to be variable when compared with other atypical antipsychotics. In addition, we previously demonstrated that the effects of aripiprazole on levels of catecholamine metabolites and plasma BDNF differed from those of other atypical antipsychotics in patients with acute schizophrenia [19,30]. Thus, these findings indicate that, unlike risperidone and olanzapine, aripiprazole does not exert dose-dependent effects on cognitive function, although its overall effect on psychiatric symptoms and cognitive functioning is equivalent to that of other atypical antipsychotics. Aripiprazole has been reported to sufficiently improve positive and negative symptoms with low incidence of sedation, weight gain, and cardiovascular risk.

However, the factors contributing to cognitive preservation in patients with schizophrenia undergoing long-term treatment with aripiprazole remain unknown. Therefore, in the present study, we evaluated biological markers, clinical data, and psychiatric symptoms in order to identify factors that influence cognitive function in this patient population.

## 2. Results

Table 1 lists the characteristics of patients included in the present study.

Multivariate analyses were performed to identify factors independently associated with cognitive function in patients with schizophrenia for each item on the brief assessment of cognition in schizophrenia Japanese version (BACS-J) and for composite scores. Brain-derived neurotrophic factor levels, number of hospitalizations, and MHPG levels were associated with verbal memory and learning. Total hospitalization period and MHPG levels were associated with working memory. Age at first hospitalization and education were associated with motor speed. The number of hospital admissions, negative subscale of the positive and negative syndrome scale (PANSS-N) scores, MHPG levels, BDNF levels, and drug-induced extrapyramidal symptoms scale (DIEPSS) scores were associated with verbal fluency. Homovanillic acid and MHPG levels, duration of illness, and PANSS-N scores were associated with attention and processing speed. Brain-derived neurotrophic factor and MHPG levels were associated with executive function (Table 2).

## 3. Discussion

The present study investigated the factors associated with preserved cognitive functioning in psychiatrically stable patients with schizophrenia treated with aripiprazole monotherapy. Interestingly, the factors affecting cognitive function differed across the various cognitive domains. The plasma BDNF level was associated with verbal memory and learning ability, verbal fluency, and executive function, which may be partly explained by dysfunction of the hippocampal complex. Brain-derived neurotrophic factor is abundant in the hippocampus, influencing memory function as well as other aspects of cognition. According to previous reports [31,32,33], the serum BDNF level is most likely associated with neurocognitive functions such as learning and memory. We also observed that plasma MHPG levels were associated with cognitive domains such as verbal learning, working memory, verbal fluency, attention and processing speed, and executive function, suggesting that preserved cognitive functioning in patients receiving aripiprazole may be partly related to activation of the noradrenergic system activation. Indeed, a previous study [22] reported that the plasma MHPG level was associated with attention and processing speed, and that eight weeks of aripiprazole treatment significantly increased plasma MHPG levels.

There are several limitations in the present study. First, it was an open-label trial rather than a double-blind, fixed-dose study. Second, the sample size was small. Third, there was no healthy control group, and the follow-up period was short. Fourth, plasma MHPG and HVA levels are thought to reflect only 30%–50% and 10%–20% of the dynamics of the brain, respectively. Fifth, the source of circulating BDNF remains unknown. Platelets, brain, and vascular endothelial cells are considered candidate sources. Furthermore, there is increasing evidence that sampling characteristics, several sociodemographic variables (such as urbanicity, age, sex), lifestyle factors (such as smoking status and food and alcohol intake), and somatic diseases are relevant determinants of peripheral BDNF levels. Thus, it is mandatory that the results presented are discussed with respect to these determinants. Finally, we did not check for potential collinearity that may exist between the different predictors; that will inflate the statistical significance unduly. Therefore, further precise and well-controlled studies considering above problems should be done to lead to robust results.

## 4. Materials and Methods

### 4.1. Patients and Procedure

The present study included 51 patients (Men/Female = 31/20) diagnosed with schizophrenia based on Diagnostic Statistical Manual of Mental Disorders, Fourth Edition, Text Revision (DSM-IV-TR) criteria (30 men, 21 women; mean age ± standard deviation (SD): 30.4 ± 7.9 years). All patients were treated with aripiprazole monotherapy in the acute stage of illness and the subsequent six months or more without any tolerability concerns. Exclusion criteria were as follows: (1) comorbid central nervous system disorder; (2) severe psychotic symptoms; (3) meeting DSM-IV criteria for alcohol or other substance dependence; (4) meeting DSM-IV criteria for mental retardation; (5) use of antidepressants; (6) treatment with electroconvulsive therapy in the six months preceding the study; and (7) inability to understand the study protocol.

Psychiatric symptoms were assessed using the PANSS. Background information including gender, age, dominant arm, age at onset, duration of untreated psychosis, number of hospitalizations, length of hospitalization, years of education, concomitant medications, and smoking history was also collected. In addition, blood levels of HVA, MHPG, and BDNF were measured.

All subjects gave their informed consent for inclusion before they participated in the study. The study was conducted in accordance with the Declaration of Helsinki, and the protocol was approved by the Ethics Committee of University of Occupational and Environmental Heatlh, Kitakyushu, Japan (Project identification code: 08-08).

### 4.2. Japanese Version of Brief Assessment of Cognition in Schizophrenia 

Cognitive function was assessed by trained psychiatrists using the Japanese version of the Brief Assessment of Cognition in Schizophrenia (BACS-J) [34]. The BACS-J has established reliability and validity and is designed to measure various aspects of cognitive function in patients with schizophrenia [34,35]. The metric includes brief assessments of verbal memory, working memory, motor speed, verbal fluency, attention and processing speed, and executive function. The primary measures from each subtest of the BACS-J were standardized by creating z-scores (the mean of healthy controls was set to zero, and the standard deviation was set to one). All data from healthy controls were obtained from a study by [36], and a composite score was calculated by averaging all z-scores for the six primary measures. The influence of age was adjusted using age-matched cohorts of controls to calculate the BACS-J z-scores for each patient in the present study.

### 4.3. Serum Brain-Derived Neurotrophic Factor, Plasma Homovanillic Acid, and 3-Methoxy-4-Hydroxyphenylglycol Level Measurements

Serum BDNF levels were measured using a BDNF Emax Immunoassay Kit (Promega, Madison, WI, USA) according to the manufacturer’s instructions. The standard curve was linear from 5 pg/mL to 5000 pg/mL. The lower limit of detection was 10 pg/mL. The recovery rate was >90%.

Levels of plasma catecholamine metabolites (HVA and MHPG) were measured using high-performance liquid chromatography. The standard curves of plasma HVA and MHPG were linear from 0.5 ng/mL to 20 ng/mL. The lower limit of detection was 0.5 ng/mL. The intra and inter-assay coefficients of variation were 7% and 6%, respectively. The recovery rate was >80%.

All fasting blood samples were obtained between 7 a.m. and 10 a.m. Two glass tube were prepared for blood sampling. One plain tube for serum, and another tube with ethylenediaminetetraacetic acid disodium salt (EDTA-2Na) for plasma. Twenty milliliter venous blood was drawn from the participants while they were in a supine position and after they had been lying at rest overnight.

### 4.4. Statistical Analysis

Multiple linear regression analysis was performed using STATA software (Stata Corp. Ltd., College Station, TX, USA). Data were expressed as the mean ± SD on the parametric distribution of the variable. To determine variables potentially predictive aripiprazole cognitive effects, multiple linear regressions (forward-stepwise selection) were performed. The BACS-J sub-scores were used as the dependent variables, while the independent variables included gender, age at onset, duration of untreated psychosis, concomitant medications, smoking history, PANSS scores, DIEPSS scores, and levels of HVA, MHPG, and BDNF. All independent variables fit a normal distribution. A *p*-value < 0.05 was considered significant.

## 5. Conclusions

In the present study, we observed a tendency for negative psychotic symptoms to correlate with cognitive dysfunction, and that certain background factors such as education, number of hospital admissions, duration of illness, and age at first hospitalization were associated with various aspects of cognitive function in patients with schizophrenia undergoing aripiprazole monotherapy. These results suggest that treatment of psychiatric symptoms and cognitive dysfunction may be improved in patients treated with aripiprazole by controlling for these contributing factors, and that improved understanding of the mechanisms underlying these associations may lead to improved functioning and quality of life in this patient population.

## Figures and Tables

**Table 1:** Demographics of this study.

Variables		Mean ± SD
Age (years)		30.4 ± 7.9
Education (years)		13.0 ± 2.4
Onset (years)		24.9 ± 6.1
PANSS-P		16.9 ± 3.9
PANSS-N		17.8 ± 2.7
PANSS-G		35.1 ± 6.7
PANSS-T		69.9 ± 1.3
BACS-J	Verbal learning	−0.92 ± 1.34
	Working memory	−0.58 ± 1.27
	Motor function	−1.62 ± 1.45
	Verbal fluency	−0.88 ± 1.31
	Attention and processing speed	−1.69 ± 1.23
	Executive function	−0.70 ± 1.91
	Composite score ^1^	−1.04 ± 1.07

PANSS-P: Positive and Negative Syndrome Scale positive subscale scores; PANSS-N: Positive and Negative Syndrome Scale negative subscale scores; PANSS-G: Positive and Negative Syndrome Scale general psychopathological symptom subscale scores; PANSS-T: Positive and Negative Syndrome Scale total scores; BACS-J: Brief Assessment of cognition in schizophrenia Japanese language version. ^1^ Calculated by averaging all z-scores for the six BACS-J primary measures.

**Table 2:** Multivariate analysis for the cognitive function treated with aripiprazole.

Variables	β	SE	*F*	*t*	*P*	*R*^2^
*Verbal learning*						
Total explanatory power						0.55
First hospitalization age	0.04	0.02	2.58	1.61	0.12	
The number of hospitalization	−0.55	0.23	5.66	−2.38	0.02	
PANSS-N	−0.13	0.09	2.28	−1.51	0.14	
PANSS-G	0.06	0.03	3.68	1.92	0.07	
MHPG	0.39	0.18	4.68	2.16	0.04	
*BDNF*	0.08	0.03	7.48	2.74	0.01	
*Working memory*						
Total explanatory power						0.48
Education	0.10	0.19	1.77	1.33	0.19	
Total hospitalization period	−0.15	−0.36	6.23	−2.50	0.02	
PANSS-N	−0.12	−0.22	2.65	−1.63	0.11	
MHPG	0.53	0.42	10.39	3.22	0.00	
*Motor function*						
Total explanatory power						0.36
Education	0.21	0.09	5.93	2.44	0.02	
First hospitalization age	−0.06	0.02	8.04	−2.84	0.01	
MHPG	0.34	0.21	2.64	1.63	0.11	
*Verbal fluency*						
Total explanatory power						0.71
The number of hospital admissions	−0.46	0.15	8.96	−2.99	0.01	
PANSS-N	−0.17	0.06	8.43	−2.90	0.01	
DIEPSS	0.27	0.10	7.51	2.74	0.01	
MHPG	0.53	0.13	15.69	3.96	0.00	
BDNF	0.06	0.02	8.28	2.88	0.01	
*Attention and processing speed*						
Total explanatory power						0.76
Duration of illness	0.06	0.02	5.45	2.34	0.03	
The number of hospitalization	−0.57	0.12	21.82	−4.67	0.00	
PANSS-N	−0.10	0.05	3.93	−1.98	0.06	
HVA	−0.33	0.10	11.80	−3.44	0.00	
MHPG	0.76	0.11	45.87	6.77	0.00	
*Executive function*						
Total explanatory power						0.36
Education	0.16	0.12	1.75	1.32	0.20	
Duration of illness	0.10	0.06	2.60	1.61	0.12	
MHPG	0.86	0.29	8.75	2.96	0.01	
BDNF	0.10	0.04	6.56	2.56	0.02	

MHPG: 3-Methoxy-4-hydroxyphenylglycol; BDNF: Brain-derived neurotrophic factor; DIEPSS: Drug-induced extrapyramidal symptoms scale; HVA: Homovanillic acid. β: Regression coefficient; SE: Standard error; *F*: Variance ratio; *t*: *t*-Values; *P*: *P-*value; *R^2^*: Coefficient of determination.
